# Development and Validation of an Objective, Passive Dietary Assessment Method for Estimating Food and Nutrient Intake in Households in Low- and Middle-Income Countries: A Study Protocol

**DOI:** 10.1093/cdn/nzaa020

**Published:** 2020-02-07

**Authors:** Modou L Jobarteh, Megan A McCrory, Benny Lo, Mingui Sun, Edward Sazonov, Alex K Anderson, Wenyan Jia, Kathryn Maitland, Jianing Qiu, Matilda Steiner-Asiedu, Janine A Higgins, Tom Baranowski, Peter Olupot-Olupot, Gary Frost

**Affiliations:** 1 Section for Nutrition Research, Department of Metabolism, Digestion and Reproduction, Imperial College London, London, UK; 2 Department of Health Sciences, Boston University, Boston, MA, USA; 3 Hamlyn Centre, Imperial College London, London, UK; 4 Department of Neurological Surgery, University of Pittsburgh, PA, USA; 5 Department of Electrical and Computer Engineering, University of Alabama, Tuscaloosa, AL, USA; 6 Department of Foods and Nutrition, University of Georgia, Athens, GA, USA; 7 KEMRI Wellcome Trust Programme, Kilifi, Kenya; 8 Department of Nutrition and Food Science, University of Ghana, Legon-Accra, Ghana; 9 Department of Pediatrics, Section of Endocrinology, University of Colorado, Anschutz Medical Campus, Aurora, CO, USA; 10 USDA/ARS Children's Nutrition Research Center, Department of Pediatrics, Baylor College of Medicine, Houston, TX, USA; 11 Mbale Clinical Research Institute, Mbale Regional Referral and Teaching Hospital, Mbale, Uganda

**Keywords:** food, nutrient, dietary, intake, assessment, undernutrition, wearable, camera, devices, household

## Abstract

Malnutrition is a major concern in low- and middle-income countries (LMIC), but the full extent of nutritional deficiencies remains unknown largely due to lack of accurate assessment methods. This study seeks to develop and validate an objective, passive method of estimating food and nutrient intake in households in Ghana and Uganda. Household members (including under-5s and adolescents) are assigned a wearable camera device to capture images of their food intake during waking hours. Using custom software, images captured are then used to estimate an individual's food and nutrient (i.e., protein, fat, carbohydrate, energy, and micronutrients) intake. Passive food image capture and assessment provides an objective measure of food and nutrient intake in real time, minimizing some of the limitations associated with self-reported dietary intake methods. Its use in LMIC could potentially increase the understanding of a population's nutritional status, and the contribution of household food intake to the malnutrition burden. This project is registered at clinicaltrials.gov (NCT03723460).

## Introduction

Undernutrition, including protein-energy and micronutrient deficiency, remains at an unacceptably high level in many low- and middle-income countries (LMIC) (1). Childhood undernutrition—stunting, wasting, and underweight—is associated with increased morbidity and mortality (2). Undernutrition in women and adolescent girls has profound immediate and long-lasting adverse consequences on the individual and future generations (3). In addition to undernutrition, the prevalence of obesity, overweight, and associated noncommunicable diseases is increasing in LMIC, especially in the urban areas, contributing to the so-called double burden of malnutrition (4). The etiology of malnutrition is diverse and somewhat complex, but dietary intake, dietary quality, and dietary pattern play major roles (5). Accurate assessment of habitual food and nutrient intake at the individual, household, and population levels and its contribution to the burden of undernutrition is important in planning programs and policies to eradicate or reduce undernutrition. However, accurate estimation of dietary intake remains problematic. Traditional methods of dietary intake assessment, such as 24-h recall (24HR), FFQs, and food records, are labor intensive, expensive, and often do not result in accurate assessment of nutritional intake due to their dependence on self-report. Specifically, these methods are highly subjective in nature and require respondents to recall all foods and drinks consumed on a previous day for 24HR or (most often) in the previous year for FFQ, leading to recall bias and erroneous conclusions. Furthermore, when keeping food records (weighed or estimated), individuals can react to the assessment by undereating or underrecording food intake (6, 7) and therefore the records might not reflect habitual intake.

Efforts have been made in high-income countries to minimize reporting bias and improve the accuracy of dietary recall and records. These include shifting from pencil-and-paper–based 24HR to computer- or web-based applications such as Intake24 (8), myfood24 (9, 10), Oxford WebQ (11), Automated Self-Administered 24HR (ASA24) (12), GloboDiet (formerly EPIC-Soft) (13, 14), and so forth. These computerized multipass 24HR apps are designed to minimize forgotten food items, improve portion size estimation, and facilitate the automatic generation of food codes. However, the use of such tools requires access to a computer, internet connectivity, computer literacy, and numeracy skills, which limit their deployability in LMIC settings. In addition, numerous studies have reported significant discrepancies between actual food intake as measured by doubly labeled water and that reported by self-administered dietary recall (6, 15–17).

Advancement in smartphone and wearable technologies provides an opportunity for collaboration between nutritionists and engineers to develop low-cost, convenient, quicker, and more accurate methods of dietary intake assessment. Mobile phone–based methods, where respondents are asked to upload images of foods and meals eaten, can be used as a stand-alone (image-based) method or to assist traditional self-reported dietary intake methods (image-assisted). Images are intended to aid in the estimation of portion sizes and in helping respondents remember the foods eaten but not reported (18). Furthermore, apps have been developed on smartphones to capture images of food plate, before and after eating, and backend software is used to estimate portion size and nutritional content (19–21). Despite the advantages of reducing administrative data handling, recall bias, and dietary underreporting, mobile phone–based methods also rely on user input, which still leaves some subjectivity of dietary data collection. In addition, using mobile phones to capture food images requires some level of training (competence), understanding of the commands set in the phone (literacy), and the mind to remember to take images of each food and meal before and after eating (burden), which might pose a significant challenge to their effective use in LMIC settings. Thus, there remains a need to overcome the limitations of self-report dietary intake assessment methods, and the limitations that prevent the deployability of some of the recent technologies in LMIC settings, in order to achieve an accurate estimate of individual- or population-level dietary intake.

The development of methods that require minimal or no user input, the so-called “passive” methods, could help break through the barriers around misreporting bias and errors associated with self-reported dietary intake methods. Such passive methods include wearable cameras and sensors that automatically capture images of food consumption and produce the nutritional analysis of the intake. Herein, we describe the protocol of a study designed to develop and validate an objective, passive, image- and sensor-based dietary assessment method for estimating food and nutrient intake in households in Ghana and Uganda.

## Methods

### Study design

This project is designed to validate an objective, passive, image- and sensor-based dietary assessment method for the estimation of dietary intake of adults and children in households in LMIC. The study uses wearable and fixed camera devices around households to capture images of food preparation and cooking, and employs automated [artificial intelligence (AI) and deep learning] and manual (visual estimation) techniques to recognize foods on the images, and estimate portion size and nutrient content. This passive, image-based method will be validated against an established method of assessment of true intake—supervised weighed food record in this case—to provide a measure of its accuracy. In addition to validation, the accuracy of the new method will be compared with interviewer-administered 24-h dietary recalls, a widely used dietary intake assessment method, in field studies in Ghana and Uganda.

### Setting and participants

The field studies for this project will be conducted in Ghana and Uganda. In each country both rural and urban/periurban communities have been selected for inclusion to facilitate the validation of the passive dietary assessment method across diverse geographical locations and cultural and community practices relating to food acquisition, preparation, consumption, and eating. In the 2 countries, households comprising a child/children aged <5 y and/or an adolescent child or children (aged 10–19 y) will be invited to participate. These age groups are of interest because of their association with marked nutritional requirement and prevalence of undernutrition. Thus, the validation of these dietary assessment tools in populations at risk of undernutrition will support future diet- and nutrition-related research in the area. In each community, local field staff and community leaders will help to identify households for recruitment and participation. Informed consent will be obtained from adults in each household, and assent from minors aged 13–17 y, before enrolment into the study. For the purpose of informed consent, trained local field staff with knowledge of the local language and culture will explain the full details of the study to the participant, covering all aspects of the study as detailed in the participant information sheet. The participant information sheet will be translated into local languages and read to illiterate participants. All participants will be given ≤48 h to decide whether they would like to participate. In addition, candidate participants will be given the opportunity to speak to an in-country study investigator or external nutrition and health personnel about the study. If a recruitee agrees to participate, written consent will be obtained through either a signature or fingerprint. Participating households will be compensated as per local ethics committee recommendation.

### Devices

**FIGURE 1:**
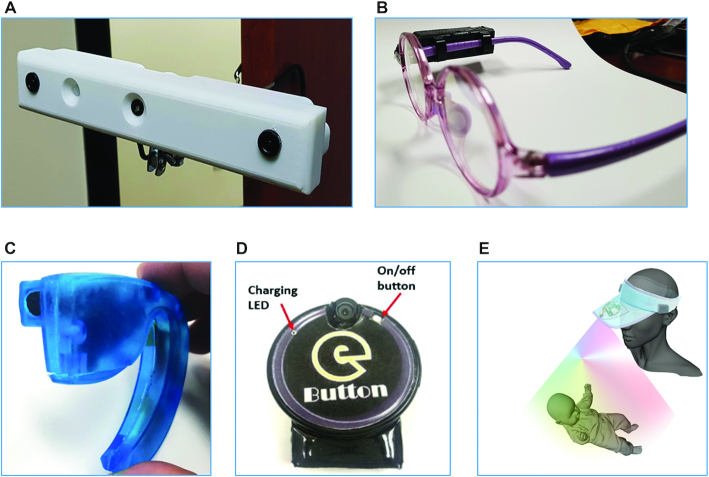
Wearable camera devices used for passive capture of images of food intake and food-related activities in the households: (A) Foodcam, (B) Automatic Ingestion Monitor (AIM), (C) Ear-worn, (D) eButton, and (E) eHAT.

To capture the feeding/eating practices across the different age groups, individual preferences, and the specific food-related activities of people in LMIC, different camera- and sensor-based devices will be integrated into a comprehensive passive dietary assessment system. This system comprises camera- and sensor-based devices for passive capture of images of food preparation and intake, a data repository for image storage, and custom software for food recognition, volume estimation, and nutritional analysis. To achieve these objectives the following camera and sensor devices (**Figure 1A–E**) will be used in the study:

- *Foodcam*—a stereoscopic camera mounted in kitchens or food preparation areas to capture images of a cooking process. The key features of Foodcam include: i) two 5-MP (megapixel) cameras with electronically switchable visible/infrared filers, enabling imaging in visible and infrared spectra; ii) an infrared structured light projector, for use in 3-dimensional image reconstruction of food items; and iii) a passive infrared motion detector, for motion sensing and auto-switch of the device. Foodcam is powered by a built-in rechargeable battery, which when fully charged can continuously capture images for ≤14 h. It has robust internal data storage capacity, allowing for ≤1 wk of continuous data collection and storage.
- *Automatic Ingestion Monitor, version 2 (AIM-2)*—an improved version of a previously described model (22). The AIM-2 is a 5-MP, rechargeable camera device attached to the temple of eyeglasses, providing a gaze-aligned capture of images. The user wears an eyeglass with the device attached during food intake, and the device captures images of food consumption every 5–15 s. The device also has a built-in accelerometer sensor for detection of food intake. Captured images and sensor data are stored on an internal secure digital (SD) card, with the capacity to store ≤3 wk of data. When fully charged, the device can continuously capture ≤20 h of image data and ≤2–3 d of sensor data.
- *Ear-worn*—a lightweight miniaturized camera device shaped like a Bluetooth headset. The built-in camera is directed outwardly, providing a good viewing angle for capturing video sequences of food intake. The device can store up to 32 GB (gigabytes) of video, and the battery, when fully charged, is adequate for capturing video events of an entire day of food intake.
- *eButton*—a circular chest-pin–like device, worn by attaching it to clothing using a needle-clip or worn using a lanyard or necklace (23). eButton is equipped with a camera of 170° angle of view with a downward tilted lens, allowing the device to record food in front of the wearer. It captures images at intervals of 3–5 s at a resolution of 1280 × 720 pixels per image. It has a battery life of 16 h, when fully charged, and the internal storage can store 1 wk of imagery data.
- *eHAT*—a camera device attached to the brim of a hat. An electronic component (including camera and SD card) similar to those of eButton is embedded underneath the front tip of the brim of a hat. This special design enables imaging of breastfeeding episodes, to provide an estimation of the frequency and duration of breastfeeding in 1 d. A mother or caregiver wears the hat during breastfeeding, and the camera, which is angled toward the chest region, takes time-lapse images every 3–5 s of the feeding. eHAT could also be used to capture images of other infant feeding practices, such as bottle-feeding or spoon-feeding of young toddlers in a situation where the infant being fed is positioned on the chest region of the mother or caregiver.

### Study procedures

To develop an objective, passive dietary assessment method to enable accurate estimation of an individual's dietary intake, the camera devices will be subjected to rigorous testing to evaluate their functionality, performance, and acceptability before undergoing a validation study in households in Ghana and Uganda. These tests are important to identify and solve technological problems at an early stage and support the deployment of devices with greater accuracy, acceptability, and applicability in LMIC settings. To this effect, 3 conjoining studies have been planned as part of this project.

#### Study 1: Evaluation of the acceptability, functionality, and relative validity of passive dietary assessment devices in estimating dietary intake in adults and children of Ghanaian and Kenyan origin living in London

The overarching aim of this study is to determine the strengths and weaknesses of the different image devices in capturing food intake. The accuracy of dietary intake estimates from the images will be assessed in comparison with weighed food records. Their acceptability will be assessed in adults and children of Ghanaian or Kenyan origin living in London, to guide the design and development of a passive method deployable in LMIC. The study will be conducted in 2 parts, 1a and 1b.

*Study 1a*. This substudy will evaluate the acceptability and functionality of the camera devices in capturing images of food intake, and the accuracy of dietary intake estimation from the images in adults in different laboratory settings, mimicking unique LMIC conditions. These conditions include using foods of Ghanaian and Kenyan origin, eating under low lighting to mimic a scenario of no or inadequate supply of electricity, and communal eating [i.e., where >1 person eats from a shared plate of food (24)]. Dietary intake estimation from the food images will be compared with weighed food records for validation of the passive dietary assessment method in adults as reported elsewhere. Eighteen adults (≥18 y old) of Ghanaian or Kenyan origin living in London, United Kingdom, will be recruited to participate. The inclusion criteria are: an adult of Ghanaian or Kenyan origin; able to eat foods typical of Ghana and Kenya; have no problem with chewing or food allergies; and able to use a wearable camera device during eating. Consenting adults will be brought to the National Institute for Health Research Clinical Research Facility (CRF) at the Hammersmith Campus of Imperial College London for the study visit activities. The participants will be divided into 3 groups of 6 people and will visit the CRF 3 times (once a week). Three devices, the eButton, AIM, and ear-worn device, will be included for testing. On each visit, a standardized weighing scale (Salter Brecknell) will be used to preweigh foods of Ghanaian and Kenyan origin. Preweighed foods will be presented to the participants to eat, and a passive camera device will be used during eating to take time-lapse images or video of their food intake. Participants will be asked to eat until full. Leftover foods will be weighed (postweight) and recorded. The net difference between pre- and postweighed foods will provide an estimate of weighed food record. During the 3 visits, participants will eat under either good lighting, low lighting, or share a plate of food with other participants. At the end of each visit, participants will be given a device assessment form and asked to provide an acceptability rating (ranging from 1 to 5) on comfort and ease of use, willingness to use a similar device in the future, and choice of a device they most prefer among the 3. Study staff at the CRF will take notes of the challenges encountered and/or reported by the participants on the placement of the devices (ergonomics). In addition, an assessor independent to the data collection will conduct an unbiased assessment of the image quality of the devices and completeness of data collection during the eating episodes to determine their functionality. Images of food intake from the devices will be downloaded onto a computer and stored in cloud storage. The stored images will be assessed for dietary intake estimation using image annotation software. Food analysis software (Dietplan7; Forestfield Software Ltd) will be used to estimate energy, macronutrients (protein, carbohydrates, and fat), and a panel of micronutrients in the weighed food portions eaten by the participants.

*Study 1b*. In the second part of the study, 11 families of Ghanaian and Kenyan origin who have children aged 0–18 y will be recruited for further testing of the devices in households in London. The purpose of this substudy is to test the abilities of Foodcam and the wearable camera devices in capturing images of food preparation and intake, respectively, to assess the acceptability of the devices in children, and the accuracy of estimation of dietary intake from the food images compared with weighed food records. Households will be recruited and enrolled through informed consent of the household head (mainly mothers), and assent of teenagers (aged 13–17 y). Consented households will be visited twice and, on each visit, a meal of Ghanaian and Kenyan origin will be cooked. At the start and during cooking, all ingredients for making the meals will be weighed and recorded, and Foodcam will be mounted in the kitchen overlooking the cooking area to take time-lapse images of the food preparation. At the end of cooking, foods served to the children will be weighed and recorded before eating, and the children will wear a camera device during eating to take images of their food intake. Children will be asked to eat until full and any leftover food will be weighed and recorded for completion of weighed food records. For families who eat using shared plates, Foodcam will be used in the dining area to record their food intake. Food images captured by the devices will be downloaded and stored in cloud storage. Finally, a device assessment form will be given to the children to provide an acceptability rating (ranging from 1 to 5) on how comfortable the devices were on their body, their ease of use, how much they interfered with their eating, and the children's willingness to use similar devices in the future. Mothers will be allowed to complete the questionnaire for younger children who are unable to provide coherent answers, and to guide older children in answering the questions, without the interference of study staff. Portion sizes and nutritional analyses will be estimated from the food images and validated against weighed food records.

The Imperial College Research Ethics Committee has provided ethics approval for these studies: approval reference numbers are 18IC4780 and 18IC4795 for the study at the CRF and at households of participants, respectively.

#### Study 2: Evaluation of the feasibility and acceptability of passive dietary assessment devices in households in Ghana and Uganda

The purpose of this study is to evaluate the feasibility, acceptability, and performance of passive assessment devices in capturing food acquisition, preparation, and intake of households in Ghana and Uganda. In addition, this study aims to collect information on household structure, food, and eating behavior through an in-depth household interview, to inform the configuration and deployment of the camera devices appropriate for the food environment and eating culture of households in Ghana and Uganda.

Twenty-eight households (14 in each country) will be recruited and enrolled into the study. Prior to the recruitment of households, comprehensive community engagement meetings will be held with the community liaison office of the host institution, area government nutrition and health offices, community elders, and members of the communities to discuss the study and the use of camera devices to capture images of household food acquisition, preparation, and intake. Concerns raised by the communities will be addressed before the recruitment of households or start of the study.

Community elders and local field staff will help in identifying households for recruitment. Households will be enrolled when they meet these inclusion criteria: *1*) has ≥1 parent (mother or father); *2*) has a child or children aged <5 y and/or adolescent child/children (aged 10–19 y); *3*) members of the household eat the same prepared meal (i.e., each member does not prepare and eat separately); and *4*) willingness to use camera devices to record food preparation and intake. Enrollment of households will be through the household head or the primary food preparer, other adults in the households, and assent of teenagers.

Trained local field staff conversant with the local dialect will visit consenting households to conduct an in-depth key informant interview with the primary food preparer. The interview will elicit an understanding of the culture around the household's choice of ingredients, common recipes, food preparation, eating practice, eating environment, and structure of the household to inform the choice or configuration of the passive dietary assessment devices to use in the household. The interviewer will ask the primary food preparer how many people live in the household, what is the household's primary food source (e.g., does the household obtain its food mainly through small-scale gardening, farming, purchase from local market, supermarket, etc.), what are the common household meals, household's choice of ingredients, common recipes, distribution of food among household members (e.g., whether a hierarchy of food distribution exists in the household), eating practice (e.g., use of individual plate for each household member or communal eating/shared plate), eating environment (i.e., where cooked food is consumed in the household), and cooking space (e.g., whether the household has a kitchen—a dedicated room for cooking and serving food—or uses an open space for cooking and serving food). All the in-depth key informant interviews will be audio-recorded in the language of choice of the participants and the transcripts transcribed in English for analysis. During the interview process, care will be taken that the activity is not too intrusive, and that the dignity and privacy of the household are maintained.

After completion of data collection on the composition, structure, and eating habits of the household, a household device deployment plan will be implemented based on the response obtained for each household. This plan will include the devices to use and their placement around the households. For example, a plan of mounting Foodcam in an open cooking area for households that do not have a built kitchen, use of devices to monitor shared plated eating for practicing households, and use of eHAT to capture images of breastfeeding episodes in households that have a breastfeeding infant. The proposed deployment plan will be discussed with members of the households before any testing. Concerns raised by household members, especially those around what is acceptable or not, will be considered.

When an agreement on the deployment of the devices within the households has been reached, testing of the feasibility and acceptability of the devices will commence. This testing will occur within a week, including a weekend day, in the following order:

*Day 1 (household training on the use of the devices)*. On this day, field staff will train household members how to wear the camera devices on their body. Each household member will be assigned only 1 device. The primary food preparer or representative will be trained how to mount the Foodcam in the kitchen or cooking area. If the household uses shared plates to eat, the members will be trained how to mount the device in the dining area of the household. The demonstration will include training on how to switch the devices on and off, how to record using the devices, and how to download the images captured by the devices. During the training session, staff will address any questions or concerns that the participants have raised. After the training, a convenient day will be agreed with the household on the next day of testing the device. Depending on the size of the household, this training could take 1–2 h.

*Day 2 (first day of recording using the devices)*. On this day, Foodcam will be used to record all the food preparation/cooking in the household. Each member of the household will be given a camera device to record his/her food intake and food-related activities for the whole day. Local field staff will visit the households in the morning to supervise mounting of the Foodcam in the kitchen area and help household members with wearing their individual devices. The field staff will stay at the household for some time (between 30 min and 1 h) to ensure all the devices are properly mounted/worn and answer any questions or concerns answered. Field staff will take extra care to ensure their presence does not cause a great deal of inconvenience to household members and does not interfere greatly with their routine. Staff will conduct spot checks around the time of consumption of the household's main meals (i.e., breakfast, lunch, and dinner) to ensure all the devices are worn correctly. Finally, staff will return to the households in the evening, after the final household meal is eaten, to gather all the devices, thank the participants for their cooperation, arrange a convenient day for the next day of testing, and return to the project office.

At the office, all the images will be downloaded, reviewed, and organized by meal. Any problems or challenges encountered during the recording will be discussed with the local study coordinator, site principal investigator, and the technology team to ensure the problems are rectified prior to the next day of testing the devices.

*Day 3 (assessment of battery life and user-friendliness of the devices)*. On this day, field staff will return to the household to test the battery life of the devices. This is important to establish whether the battery life in the field testing matches that of the manufacturer's expectation. The staff will also monitor the use of the devices by the participants. This involves assessing the ability of the participants to switch the devices on and off, recharge the devices using a portable charger, and connect to a laptop to download the images captured from the previous day of testing. This is to ensure families have full confidence in the devices and hopefully minimize any potential doubts about their use in the households. The staff will also address any problems encountered during data collection on the previous day and make sure household members are ready for using the device on the next day.

*Day 4 (second day of recording using the device)*. On this day, field staff will return to the household in the morning to provide a clean set of devices to use for that day and supervise their mounting and installation. The same procedure described above in Day 2 of testing will be followed. On this day, however, household members (except breastfed infants) will be assigned an individual wearable device different from the 1 they used previously, to ensure everyone has a feel of the different devices to help in the evaluation of the device's acceptability.

Finally, a field staff member different from those interacting with the households in the 4-d period will visit the households. S/he will present household members with a brief questionnaire to determine the acceptability of the devices among the study participants. The questionnaire will ask the household members to rate (from 1 to 5) the specific characteristic of each device such as ease of use, convenience on the body, how much it interferes with their eating and daily routine, how much they would like to use the device in the future, and their preferred choice of device. This questionnaire should take 10–15 min to complete.

The following criteria will be used to assess the feasibility, performance, and reliability of the passive dietary assessment devices:

- Reliably records image/sensor data in the field with no or minimal missing data.
- Provides information to enable accurate identification of food, portion size estimation, and conversion to nutrients—i.e., sufficient image/data quality for a nutritionist to estimate nutritional intake.
- Usability—i.e., the device is user friendly and can be used by a nonengineer.
- Battery life—sufficient duration for the data collection session.
- Minimal recharge time—does not require any recharge during use or at most, only once.
- Comfortable and convenient to wear—no irritation to the wearer and does not disrupt their daily activities.
- Installation—easy to install/setup by household members in the field.
- Suitable for most households—i.e., device is rated at least average or above for acceptability (i.e., mean rating ≥3) from household members.

This phase of the study is crucial because it will inform whether the devices are of good acceptability and performance rating before proceeding to the validation study. In the event that the acceptability and performance of the devices are poor, we will work to improve the devices and/or the factors responsible for the poor performance to ensure the devices and conditions for testing are good enough to conduct a validation study.

#### Study 3: Assessment of the validity of passive assessment method in estimating dietary intake in households in Ghana and Uganda

The purpose of this study is to test the validity of passive dietary assessment method relative to a weighed food record, in estimating an individual's dietary intake. The weighed food record is used as the gold standard method in this study. A tablet-based, interviewer-administered multiple-pass 24-h dietary recall will also be performed for comparison. Portion size picture aids will be used in the 24-h dietary recall.

Local field staff and community leaders will help in identifying 88 households (44 in each country) for enrollment in this study. The criteria for inclusion are the same as those described for Study 2. Foodcam will be mounted in the household's kitchen or cooking area to capture images of food preparation. Household members will be given a camera device to record their whole-day (waking hours) food intake, and the captured images will be used in the estimation of their dietary intake. However, only 2 randomly selected index cases (an adult and a child) in each household will have their corresponding dietary intake assessed using weighed food records and 24-h dietary recall. Dietary intake data from the devices and the 24-h dietary recall will be compared with weighed food records for accuracy evaluation. Prior to using the devices in the households, a questionnaire (similar to that used in Study 2) will be used to understand the structure, composition, and eating habits of the households to help implement a device deployment plan, especially for those who were not recruited in Study 2. To help recognize and effectively manage the data collection and logistical challenges that might arise in this study, this validation study will be conducted in 2 phases:

*Phase 1: Pilot testing of the validation protocol in a sample population*. This phase is aimed at testing the study protocol in a sample of the households to help identify and address challenges that might arise during the data collection process. Study teams in the 2 countries will recruit 8 households each (16 households in total) for this purpose. The camera devices will be set up to record food preparation and intake in the households for a day. On the same day, the meals of the 2 index cases in each household will be weighed before and after eating. On the next day, a staff member not involved in the data collection on the previous day will visit the households to complete the multiple-pass 24-h dietary recalls—a method comprising multiple and sequential layers of questioning to increase accuracy in the reported food intake. The primary caregiver will complete the recall data for smaller children (especially those aged <5 y).

Data from the devices, weighed food record, and 24-h recall will be uploaded onto the cloud-based study database. These data will help determine the fidelity of the study protocol, robustness of the image capture and data transfer, identify challenges, and help inform any potential amendments to the protocol before the validation study in the target population.

*Phase 2: Validation in the target population*. In this phase, the passive dietary assessment devices will be validated in 88 households in the 2 countries. The devices will be set up to record food preparation and intake in the recruited households for 3 nonconsecutive days, preferably 2 weekdays and a weekend day. On each day, study staff will visit the households in the morning to mount the Foodcam in the kitchen/cooking area and help members of the household wear their devices. The devices will record an individual's food intake for the whole day during waking hours. At the end of each day, staff will return to the households to collect all the devices. In addition, the same staff will weigh and record the food intakes, before and after eating, of the index cases for the weighed food record measurement. After each day, a different staff member will visit the households to complete the 24-h dietary recall for the index cases.

The dietary data collected from the households will be taken to the project office, where the images will be downloaded, reviewed, organized by meal, and uploaded onto the study database. Custom software (described below) will be used to identify the foods on the images, and estimate the portion size eaten and its nutritional content. Weighed food record and 24HR data will be analyzed by independent staff using nutritional analysis software (Dietplan) to estimate the nutritional content. The dietary intake from the images and 24HR will be compared with the weighed food record for accuracy evaluation.

The ethics committees (Institutional Review Boards) of University of Georgia, USA, and Noguchi Memorial Institute for Medical Research, Ghana, have approved Studies 2 and 3 for Ghana (approval reference: STUDY00006121 and IRB00001276, respectively). Studies 2 and 3 in Uganda have been approved by Mbale Regional Referral Hospital Research Ethics Committee (MRRH-REC) and the Uganda National Council for Science and Technology (UNCST) (approval reference: MRRH-REC OUT0051/2019 and HS2680, respectively).

### Assessment of dietary intake

**FIGURE 2:**
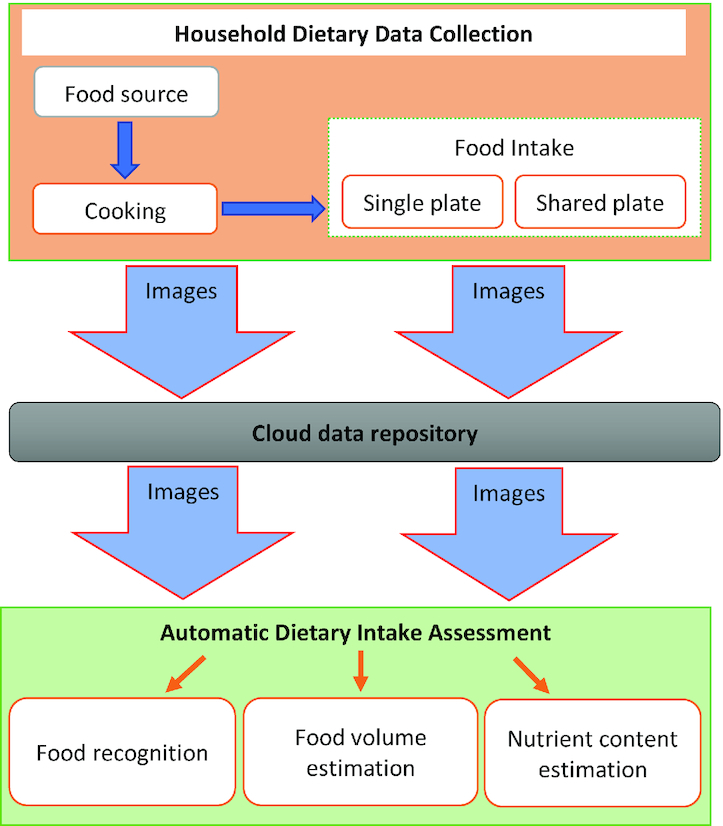
A schematic diagram of the study plan. Wearable and fixed camera devices will be used to collect images of food intake and related activities such as cooking in households in Ghana and Uganda. Images captured by the devices will be stored in cloud storage, and the stored food images will be used for food recognition and estimation of portion size and nutrient content, thus providing objective, passive dietary assessment.

This study aims to develop an objective, passive method for accurate estimation of an individual's food and nutrient intake. To facilitate this, the study will explore different image analysis methods to help develop reliable, accurate, and cost-effective backend software capable of automatic food recognition, estimation of portion size, and nutritional content of foods on images captured by the devices in the households (**Figure 2**). The different methods proposed for achieving these nutritional outcomes are described below.

#### Food image recognition

The convolutional neural network (CNN) method, a start-of-the-art deep learning technique used in image recognition (25), will be used to enable automatic recognition of images of foods captured in this study. CNN reveals intricate structures/features on input data (e.g., pixel value of an image) through transforming the raw data into a suitable internal representation, which the learning subsystem can detect and classify. Images of foods consumed in Ghana and Uganda will be collected for the creation of a food image dataset, and to train the neural network to recognize the food on the images. CNN will be validated against nutritionist-annotated images to determine its accuracy, before its application on the actual study food images.

#### Portion size estimation

This study will use the images of foods captured by the devices to quantify the amount of each food an individual consumed in a day. This will be achieved using the following methods.

##### Visual estimation

This method relies on a trained eye to estimate visually the quantity of food on images. To do this accurately, the assessor should be familiar with the local foods as well as having knowledge of the use of aids for portion size estimation. In this study, dedicated local staff in Ghana and Uganda will be trained to perform the visual portion size estimation. Training will consist of: *1*) learning the method of estimation; *2*) practicing the estimation on a standard practice deck of images with known portion sizes of food; and *3*) testing on a selection of images not included in the practice deck. Each assessor will be retested every 3–6 mo to track consistency and accuracy, and additional practice will be conducted if needed before retesting.

**FIGURE 3:**
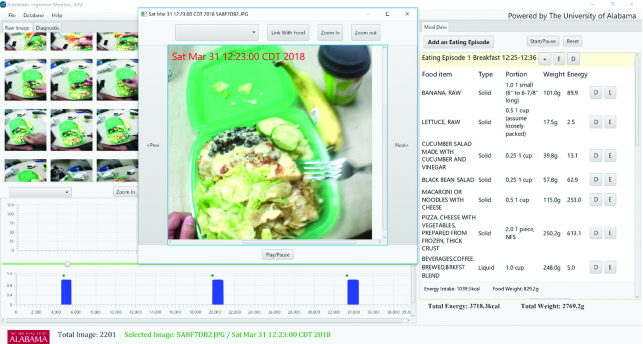
The Automatic Ingestion Monitor (AIM) software—an image annotation and dietary intake assessment software. AIM distinguishes between eating (blue bars, bottom left) and noneating episodes. Clicking on the blue bar displays images from the detected eating event. The images can be browsed individually and magnified to provide a good view of all the foods on the image. The software supports the incorporation of standard food composition databases. The software also supports visual estimation of portion sizes.

Food images for each eating occasion will be viewed using custom software, the AIM software (**Figure 3**). The portion size for each food will be estimated from images captured at the beginning and end of each eating occasion. However, all images for each entire eating occasion will be viewed to determine if additional portions of food were added to or subtracted from the plate or bowl before the end of the eating occasion, and if so the portion size of the additions and subtractions will also be estimated and taken into account to compute total consumption. An example of an addition would be a person taking a second helping; an example of a subtraction would be a person sharing the food on their plate with another person (e.g., a mother feeding a toddler from a common plate). A combination of portion size aids and comparison of food with common reference objects will be used to estimate portion sizes. The reference objects will include serveware (plates, bowls, cups, eating utensils, etc.) and hands/fingers appearing in images next to the foods and beverages. Foods not directly estimated in grams or kilograms will be estimated in volume (e.g., milliliters) and converted to grams using an estimated density (grams per milliliter) from INFOODS (International Network of Food Data Systems) (26, 27) and other sources such as USDA Food Composition Databases.

##### Mathematical model

This method estimates food volume using a mathematical model/equation. This model relies on the measurements (height and diameter/width) of serveware (i.e., cups, bowls, plates, basins etc.) and cookware (i.e., pot and pans) used in the household, as a reference scale. To obtain the reference scale, field staff in the 2 countries will arrange to visit the households to measure and record the height and width of the serveware household members use to eat. Image captured by a wearable device at the beginning and end of an eating episode will be used to estimate the volume of food consumed using the formula:

(1) $$\begin{eqnarray*}
V = \frac{{\pi kl{d^2}h}}{4}
\end{eqnarray*}
$$

where *V* represents the estimated volume, *k* is the shape parameter, *l* is the level parameter, *d* is the diameter/width of the serveware, and *h* is the height of the serveware. The shape parameter (*k*) can be obtained using 2 methods. In the first method (the precise method), the serveware (e.g., a bowl) is filled with water to its brim to measure and record (in grams) the net weight of water the serveware contains. The parameter *k* is then obtained by dividing the net weight of water [which has the same value in grams as the volume in milliliters (i.e., 1 g ≡ 1 mL)] in the serveware by the calculated volume (also in milliliters) of a hypothetical cylinder having the same diameter and height as the serveware. In the second, which is the approximate method, *k* is selected empirically from a range between 0.5 and 0.8, depending the shape of the serveware. The level parameter (*l*), which represents the fill level of bowl or cup, is visually estimated from the captured image. In this method, *l* must be chosen under the condition 0 < *l* ≤ 1, where 0 and 1, respectively, correspond to “completely empty” and “completely full.”

##### Computational estimation

In this method, a combination of ellipse extraction algorithm, deep learning, and AI will be used to estimate food volume in different serveware such as bowls, cups, basins, and plates.

Generally, in imaging, the circular edge of serveware such as bowls and cups appears elliptical on images because the lens of a wearable camera is usually at a certain angle (∼40°) to the top edge of the serveware. In this study, we will use a custom-made ellipse extraction algorithm to carefully extract the elliptical outline of serveware on the images, overcoming a number of challenges; these include partial obscuring of the elliptical outline by food, and multiple ellipses due to circular food patterns or printed decorations on the serveware. When the desired elliptical outline of the serveware is extracted, the eccentricity of the ellipse, the measured diameter of the container, and several parameters of the camera (called intrinsic parameters) are utilized to construct a coordinate transformation from the image pixel coordinates to the real-world coordinates. This transformation is a key step in enabling food volume estimation in a metric unit (e.g., milliliters) (28, 29).

For foods served on plates, we have previously explored the use of fitting a computer-generated net cover (wireframes) of preselected shapes (e.g., a sphere or a wedge) to the food in an image to aid in the estimation of food volume (30). Although successful, this manual process is time consuming and unsuitable for a large-scale study. In addition, the wireframe method is imprecise when the food shape is highly irregular, which is common with semisolid, amorphous foods. For the proposed study, we have developed an advanced method using an AI technology. Standard volumetric reference measures such as a cup, a golf ball, a fist, a deck of cards, and so forth were used to train the AI system to estimate the volume of food on images, akin to how a human is trained to conduct visual portion size estimation. The CNN in the AI system takes the food images as the input and a set of probabilities with respect to the standard volumetric references is produced as output, and the probability values are then combined to yield the estimated food volume. Preliminary testing of the AI system in a laboratory setting across 630 food images of known volume produced an average relative error of 11.5% (M Sun, W Jia, ML Jobarteh, unpublished results, 2019). This system will be further enhanced and used in the proposed study.

#### Assessment of nutritional content

Nutritional content of foods captured on the images will be assessed using the AIM software as the interface (Figure 3). The AIM software is capable of incorporating different food composition databases, such as the West African Foods database (31), Kenyan Food database (32), UK Integrated Food Composition dataset (33), and the USDA Food Composition databases (34), allowing users to choose from databases. In addition, the AIM software also accepts ad hoc databases, for example, in situations where “back of pack” nutrient data of food brands need to be added. The closest match for foods in this study will be selected, preferentially the West African Foods database for Ghanaian foods and the East African Food database for Ugandan foods. When required these selections will be supported by other food composition databases, such as the USDA and UK databases. The nutrition analysis for each person's daily food intake will be saved and exported to an Excel (Microsoft) file for analysis.

### Other assessments

#### Estimation of an individual's food intake during shared plate eating

Shared plate eating is a reality in many LMIC, and yet there are currently no methods available to accurately estimate the food and nutrient intake of individuals in that setting. This contributes to a large nutritional data gap. In this study, in addition to using Foodcam, we will employ a vision-based approach, which integrates face recognition, hand detection, and food recognition, using a 360° camera (35). A 360° camera will be set up in selected households where shared plate eating is practiced to capture video sequences of the eating. The use of a 360° camera reduces the burden of setting up multiple cameras to capture the eating episode. The vision-based approach uses deep learning techniques to recognize faces, detect hand movement and food items on a plate/bowl, and relate the food items consumed to an individual within a group. It can then estimate each individual's food intake by analyzing the hand–food interactions and the variation of hand–face distance of each individual throughout the eating.

#### Assessment of food acquisition and other food-related activities

Continuous, passive capture of images during the day provides a window into the daily activities of an individual. These activities could include food sourcing, food storage, preparation, cooking, eating, and feeding other household members. This study will not be using devices with GPS (Global Positioning System) functions due to concerns of land ownership/grabbing in some communities. However, the sequence of images captured by the devices during the course of the study is enough to provide information about where individuals in the households are obtaining their daily foods, such as buying from local markets, obtaining from household farm/garden, using food stocks in the household storage facilities, and so forth. The AIM software can collate all the images captured in a day and annotate them for the various food-related activities. In addition, an AI technology (36) that sequences images captured in a day (>10,000 images per individual per day) will also be used in this study. The images captured will be fed into commercial image labeling AI software (Clarifai). For each image, the AI software produces 20 words to describe what is on the image. We will then utilize a statistical analysis program to determine the activity on the image (i.e., food consumption or other food-related activities such as shopping, etc.). Our initial experiments on AI detection of food intake and related activities such as shopping and food preparation showed an overall accuracy and specificity of 91.5% and 86.4%, respectively, based on 3900 images (M Sun, W Jia, ML Jobarteh, unpublished results, 2019).

### Analytical plan

The data analysis plan will be based on comparison of dietary intake estimated using weighed food records, 24-h dietary recall, and the passive dietary assessment method. This will be achieved using the concordance correlation coefficient (CCC) of Lin et al. (37) to assess the extent of agreement in portion size estimation, daily intakes of energy, and macronutrient (carbohydrate, protein, fat, and selected fatty acids) and micronutrient (iron, zinc, vitamins A, B, D, iodine, folate, and calcium) estimates between methods. These comparisons will include the extent of agreement between the passive method compared with weighed food records, and 24-h dietary recall compared with weighed food records. A 95% CI will be calculated for each Lin CCC. The bootstrap method will be used to test whether the Lin CCC between weighed food records and the passive method is different from that between the weighed food records and 24-h dietary recall methods. In addition, Bland–Altman analysis will be used to assess the limits of agreement and examine trends in differences between methods as a function of magnitude of the value, and paired *t* tests will be used to compare the overall bias between method pairs. The passive and 24-h recall methods will be considered accurate when their estimates of nutrient intake are within ±10% of the intake value measured using weighed food records. The analyses will be conducted among the index cases and then stratified by child and adult, rural and periurban/urban, respectively. To explore factors related to the accuracy of the passive and 24-h recall methods compared with weighed food records, absolute or relative deviation, as appropriate, from the weighed food method (gold standard) will be used as an outcome and be analyzed using multiple linear regression (MLR). Candidate predictors in this MLR analysis include demography, education, urban/rural, child or adult.

Currently, we do not have an analysis plan for validation of the data on breastfeeding frequency and duration captured by the eHAT device because there are no comparable analytical methods. However, the use of the device in this study will help us to understand its acceptability and challenges, and help to inform future studies.

## Discussion

This article describes the protocol of a study designed to assess whether passive dietary intake assessment devices could be used to provide accurate and objective estimates of individual-level dietary intake data in populations at risk of malnutrition. These wearable sensor- and camera-based devices take time-lapse images of food intake passively, and custom software is used to analyze the captured images for dietary intake estimation, thus providing an objective measure. The article describes the proposed devices, their testing procedures, and the use of custom software for nutritional analysis. The study is made possible by the collaboration of nutritionists and engineers and takes advantages of technological advances that make the collection and analysis of data by wearable cameras and associated software possible.

We are validating a comprehensive dietary assessment tool that requires no self-report to assess food preparation and intake in households in Africa. This tool will be validated across different age groups and demographics to take into account the different dietary habits and challenges in accurately assessing dietary intake in different populations. In addition, this study will develop an innovative method of assessing an individual's dietary intake during shared plate eating. Shared plate eating is common in many communities in LMIC but very few methods are available to accurately assess dietary intake of individuals involved in the practice.

Despite the well-intended purpose of using the wearable cameras for dietary data capture, we are aware of the ethical implications of using such devices in households. We have thus taken steps to ensure that the privacy and dignity of participants are maintained at all times during the study. These steps include the inclusion of an image recognition algorithm that distinguishes between food and nonfood images, and the subsequent deletion of all nonfood images in our database. The Foodcam and AIM devices require a special software, only available to study staff, to start the devices and retrieve stored data for added security; this is, however, not available to Ear-worn, eButton, and eHAT. In addition, study participants will be given the opportunity to review the images after collection and can delete any image they feel uncomfortable about sharing.

In conclusion, our study intends to provide low-cost, objective, fast, and accurate methods of collecting and analyzing dietary intake data in populations in LMIC.

## Notes

This research project was funded by the Bill & Melinda Gates Foundation (ID OPP1171395).

Author disclosures: The authors report no conflicts of interest.

Abbreviations used: AI, artificial intelligence; AIM, Automatic Ingestion Monitor; CCC, concordance correlation coefficient; CNN, convolutional neural network; CRF, Clinical Research Facility; LMIC, low- and middle-income countries; MLR, multiple linear regression; MP, megapixel; SD, secure digital; 24HR, 24-h recall.
